# (*Z*)-*N*-[(*Z*)-3-(2,4-Dimethyl­phenyl­imino)­butan-2-yl­idene]-2,4-dimethyl­aniline

**DOI:** 10.1107/S1600536811053244

**Published:** 2011-12-17

**Authors:** Jianchao Yuan, Chengping Miao, Weibing Xu, Bingnian Yuan

**Affiliations:** a Key Laboratory of Eco-Environment-Related Polymer Materials of the Ministry of Education, Key Laboratory of Polymer Materials of Gansu Province, College of Chemistry & Chemical Engineering, Northwest Normal University, Lanzhou 730070, People’s Republic of China

## Abstract

The asymmetric unit of the title compound, C_20_H_24_N_2_, contains one half -mol­ecule which exhibits a crystallographically imposed center of symmetry. The benzene rings are inclined to the 1,4-diaza­butadiene mean plane by 78.3 (2)°.

## Related literature

The title compound was synthesized as a α-diimine ligand for Ni^II^-α-diimine olefin polymerization catalysts. For applications of α-diimine ligands, see: Johnson *et al.* (1995 ▶); Killian *et al.* (1996 ▶). For the design and synthesis of new α-diimine derivatives, see: Yuan *et al.* (2005 ▶); Popeney & Guan (2005 ▶, 2010 ▶); Popeney *et al.* (2011 ▶). The crystal structures of Re and Ni complexes with the title ligand were reported by Kia *et al.* (2005 ▶) and Yuan *et al.* (2011 ▶), respectively.
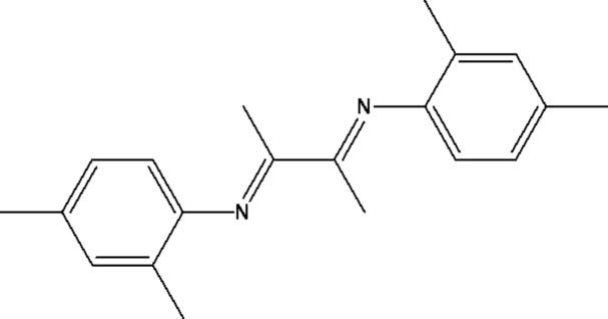


## Experimental

### 

#### Crystal data

C_20_H_24_N_2_
*M* *_r_* = 292.41
Orthorhombic, 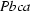

*a* = 13.50 (1) Å
*b* = 7.571 (6) Å
*c* = 16.738 (12) Å
*V* = 1711 (2) Å^3^
*Z* = 4
Mo *K*α radiation
μ = 0.07 mm^−1^
*T* = 296 K
0.23 × 0.20 × 0.14 mm

#### Data collection

Bruker APEXII CCD diffractometer
Absorption correction: multi-scan (*SADABS*; Bruker, 2008 ▶) *T*_min_ = 0.985, *T*_max_ = 0.991
5143 measured reflections
1592 independent reflections
1043 reflections with *I* > 2σ(*I*)
*R*_int_ = 0.031

#### Refinement

*R*[*F* ^2^ > 2σ(*F* ^2^)] = 0.052
*wR*(*F* ^2^) = 0.175
*S* = 1.05
1592 reflections
104 parameters
H-atom parameters constrained
Δρ_max_ = 0.21 e Å^−3^
Δρ_min_ = −0.15 e Å^−3^

### 

Data collection: *APEX2* (Bruker, 2008 ▶); cell refinement: *SAINT* (Bruker, 2008 ▶); data reduction: *SAINT*; program(s) used to solve structure: *SHELXS97* (Sheldrick, 2008 ▶); program(s) used to refine structure: *SHELXL97* (Sheldrick, 2008 ▶); molecular graphics: *SHELXTL* (Sheldrick, 2008 ▶); software used to prepare material for publication: *SHELXTL*.

## Supplementary Material

Crystal structure: contains datablock(s) I, global. DOI: 10.1107/S1600536811053244/cv5204sup1.cif

Structure factors: contains datablock(s) I. DOI: 10.1107/S1600536811053244/cv5204Isup2.hkl

Supplementary material file. DOI: 10.1107/S1600536811053244/cv5204Isup3.cml

Additional supplementary materials:  crystallographic information; 3D view; checkCIF report

## Figures and Tables

**Fig. 1.**
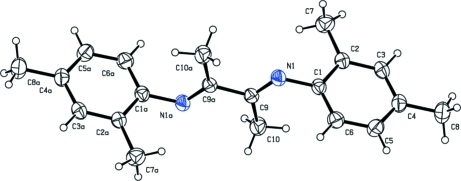
The molecular structure of the title compound, with the atom-labelling scheme [symmetry code: (a) 1 - x, 2 - y, 1 - z]. Displacement ellipsoids are shown at the 30% probability level.

## supplementary crystallographic information

### Comment

α-Diimine ligand nickel catalysts greatly attracted attention due to their high catalytic activity in ethylene polymerization (Johnson *et al.*, 1995; Killian *et al.*, 1996). Design and synthesis of the ligands is crucial (Popeney *et al.*, 2005, 2010, 2011; Yuan *et al.*, 2005). Herewith we present the title compound (I).

In (I) (Fig. 1), the single C—C bond in 1,4-diazabutadiene fragment is *trans*-configured and situated on inversion center. The dihedral angle between the benzene ring and 1,4-diazabutadiene plane is 78.3 (2)°. However, the *trans*-configured ligand can be transformed into *cis*-configured ligand in order to facilitate the formation of α-diimine-metal complexes, for examples, see  Yuan *et al.* (2011) for Ni complex, and Kia *et al.* (2005) for Re complex.

### Experimental

Formic acid (1 ml) was added to a stirred solution of 2,3-butanedione (0.052 g, 0.6 mmol) and 2,4-dimethylaniline (0.144 g, 1.2 mmol) in methanol (30 ml). The mixture was refluxed for 24 h, then cooled and the precipitate was separated by filtration. The solid was recrystallized from ethanol/dichloromethane (*v*/*v* = 8:1), washed and dried under vacuum. Yield: 0.160 g (82%). Crystals suitable for X-ray structure determination were grown from a solution of the title compound in a mixture of cyclohexane/dichloromethane (1:2, *v*/*v*).

### Refinement

All hydrogen atoms were placed in calculated positions with C—H distances of 0.93 and 0.96 Å for aryl and methyl type H-atoms, respectively. They were included in the refinement in a riding model approximation, with *U*iso = 1.2-1.5 *U*_eq_(C).

### Crystal data

**Table:**

C_20_H_24_N_2_	*D*_x_ = 1.135 Mg m^−^^3^
*M**_r_* = 292.41	Mo *K*α radiation, λ = 0.71073 Å
Orthorhombic, *P**b**c**a*	Cell parameters from 1144 reflections
*a* = 13.50 (1) Å	θ = 2.9–23.2°
*b* = 7.571 (6) Å	µ = 0.07 mm^−^^1^
*c* = 16.738 (12) Å	*T* = 296 K
*V* = 1711 (2) Å^3^	Block, yellow
*Z* = 4	0.23 × 0.20 × 0.14 mm
*F*(000) = 632	

### Data collection

**Table:**

Bruker APEXII CCD diffractometer	1592 independent reflections
Radiation source: fine-focus sealed tube	1043 reflections with *I* > 2σ(*I*)
graphite	*R*_int_ = 0.031
φ and ω scans	θ_max_ = 25.5°, θ_min_ = 2.4°
Absorption correction: multi-scan (*SADABS*; Bruker, 2008)	*h* = −8→16
*T*_min_ = 0.985, *T*_max_ = 0.991	*k* = −6→9
5143 measured reflections	*l* = −16→20

### Refinement

**Table:**

Refinement on *F*^2^	Secondary atom site location: difference Fourier map
Least-squares matrix: full	Hydrogen site location: inferred from neighbouring sites
*R*[*F*^2^ > 2σ(*F*^2^)] = 0.052	H-atom parameters constrained
*wR*(*F*^2^) = 0.175	*w* = 1/[σ^2^(*F*_o_^2^) + (0.0962*P*)^2^ + 0.2091*P*] where *P* = (*F*_o_^2^ + 2*F*_c_^2^)/3
*S* = 1.05	(Δ/σ)_max_ < 0.001
1592 reflections	Δρ_max_ = 0.21 e Å^−^^3^
104 parameters	Δρ_min_ = −0.15 e Å^−^^3^
0 restraints	Extinction correction: *SHELXL97* (Sheldrick, 2008), Fc^*^=kFc[1+0.001xFc^2^λ^3^/sin(2θ)]^-1/4^
Primary atom site location: structure-invariant direct methods	Extinction coefficient: 0.009 (4)

### Special details

**Table:**

Geometry. All e.s.d.'s (except the e.s.d. in the dihedral angle between two l.s. planes) are estimated using the full covariance matrix. The cell e.s.d.'s are taken into account individually in the estimation of e.s.d.'s in distances, angles and torsion angles; correlations between e.s.d.'s in cell parameters are only used when they are defined by crystal symmetry. An approximate (isotropic) treatment of cell e.s.d.'s is used for estimating e.s.d.'s involving l.s. planes.
Refinement. Refinement of *F*^2^ against ALL reflections. The weighted *R*-factor *wR* and goodness of fit *S* are based on *F*^2^, conventional *R*-factors *R* are based on *F*, with *F* set to zero for negative *F*^2^. The threshold expression of *F*^2^ > σ(*F*^2^) is used only for calculating *R*-factors(gt) *etc*. and is not relevant to the choice of reflections for refinement. *R*-factors based on *F*^2^ are statistically about twice as large as those based on *F*, and *R*- factors based on ALL data will be even larger.

### Fractional atomic coordinates and isotropic or equivalent isotropic displacement parameters (Å^2^)

**Table:**

	*x*	*y*	*z*	*U*_iso_*/*U*_eq_	
C1	0.60082 (15)	0.9674 (3)	0.65918 (13)	0.0471 (6)	
C2	0.69975 (15)	0.9165 (3)	0.66744 (13)	0.0464 (6)	
C3	0.72926 (16)	0.8473 (3)	0.74028 (13)	0.0528 (6)	
H3	0.7946	0.8104	0.7460	0.063*	
C4	0.66613 (18)	0.8305 (3)	0.80502 (13)	0.0548 (6)	
C5	0.56961 (18)	0.8859 (3)	0.79551 (14)	0.0596 (7)	
H5	0.5257	0.8782	0.8382	0.072*	
C6	0.53728 (17)	0.9527 (3)	0.72328 (15)	0.0579 (7)	
H6	0.4717	0.9882	0.7178	0.070*	
C7	0.77174 (18)	0.9360 (3)	0.59996 (15)	0.0669 (8)	
H7A	0.7403	0.9026	0.5508	0.100*	
H7B	0.8280	0.8612	0.6092	0.100*	
H7C	0.7931	1.0567	0.5966	0.100*	
C8	0.7013 (2)	0.7515 (4)	0.88279 (14)	0.0795 (9)	
H8A	0.7642	0.6951	0.8747	0.119*	
H8B	0.6540	0.6659	0.9011	0.119*	
H8C	0.7081	0.8432	0.9220	0.119*	
C9	0.51658 (15)	0.9537 (3)	0.53705 (12)	0.0467 (6)	
C10	0.48786 (19)	0.7641 (3)	0.54753 (15)	0.0662 (7)	
H10A	0.5191	0.7177	0.5946	0.099*	
H10B	0.5088	0.6976	0.5017	0.099*	
H10C	0.4172	0.7555	0.5530	0.099*	
N1	0.56812 (12)	1.0428 (2)	0.58600 (11)	0.0516 (6)	

### Atomic displacement parameters (Å^2^)

**Table:**

	*U*^11^	*U*^22^	*U*^33^	*U*^12^	*U*^13^	*U*^23^
C1	0.0501 (13)	0.0426 (12)	0.0487 (13)	−0.0029 (9)	−0.0084 (10)	0.0010 (10)
C2	0.0488 (13)	0.0429 (12)	0.0476 (13)	0.0017 (9)	−0.0048 (9)	−0.0027 (10)
C3	0.0475 (12)	0.0526 (13)	0.0583 (14)	0.0050 (10)	−0.0116 (10)	0.0000 (11)
C4	0.0658 (15)	0.0504 (14)	0.0483 (14)	−0.0026 (12)	−0.0108 (11)	0.0014 (11)
C5	0.0630 (15)	0.0636 (16)	0.0523 (14)	0.0035 (12)	0.0046 (11)	0.0049 (12)
C6	0.0482 (12)	0.0610 (16)	0.0646 (15)	0.0054 (11)	−0.0008 (11)	0.0083 (12)
C7	0.0624 (15)	0.0685 (17)	0.0697 (16)	0.0067 (12)	0.0087 (12)	0.0054 (13)
C8	0.0911 (19)	0.089 (2)	0.0584 (16)	−0.0039 (15)	−0.0191 (14)	0.0118 (15)
C9	0.0401 (11)	0.0499 (14)	0.0501 (13)	0.0004 (9)	−0.0031 (9)	0.0045 (10)
C10	0.0783 (17)	0.0551 (15)	0.0652 (16)	−0.0118 (12)	−0.0161 (12)	0.0118 (12)
N1	0.0503 (11)	0.0505 (11)	0.0540 (12)	−0.0017 (8)	−0.0086 (9)	0.0083 (9)

### Geometric parameters (Å, °)

**Table:**

C1—C6	1.378 (3)	C7—H7A	0.9600
C1—C2	1.397 (3)	C7—H7B	0.9600
C1—N1	1.421 (3)	C7—H7C	0.9600
C2—C3	1.385 (3)	C8—H8A	0.9600
C2—C7	1.497 (3)	C8—H8B	0.9600
C3—C4	1.384 (3)	C8—H8C	0.9600
C3—H3	0.9300	C9—N1	1.269 (3)
C4—C5	1.378 (3)	C9—C9^i^	1.494 (4)
C4—C8	1.509 (3)	C9—C10	1.497 (3)
C5—C6	1.381 (3)	C10—H10A	0.9600
C5—H5	0.9300	C10—H10B	0.9600
C6—H6	0.9300	C10—H10C	0.9600
			
C6—C1—C2	119.7 (2)	H7A—C7—H7B	109.5
C6—C1—N1	120.67 (19)	C2—C7—H7C	109.5
C2—C1—N1	119.5 (2)	H7A—C7—H7C	109.5
C3—C2—C1	117.8 (2)	H7B—C7—H7C	109.5
C3—C2—C7	121.0 (2)	C4—C8—H8A	109.5
C1—C2—C7	121.2 (2)	C4—C8—H8B	109.5
C4—C3—C2	123.1 (2)	H8A—C8—H8B	109.5
C4—C3—H3	118.4	C4—C8—H8C	109.5
C2—C3—H3	118.4	H8A—C8—H8C	109.5
C5—C4—C3	117.6 (2)	H8B—C8—H8C	109.5
C5—C4—C8	121.2 (2)	N1—C9—C9^i^	116.8 (2)
C3—C4—C8	121.2 (2)	N1—C9—C10	125.18 (19)
C4—C5—C6	120.7 (2)	C9^i^—C9—C10	118.0 (2)
C4—C5—H5	119.6	C9—C10—H10A	109.5
C6—C5—H5	119.6	C9—C10—H10B	109.5
C1—C6—C5	121.0 (2)	H10A—C10—H10B	109.5
C1—C6—H6	119.5	C9—C10—H10C	109.5
C5—C6—H6	119.5	H10A—C10—H10C	109.5
C2—C7—H7A	109.5	H10B—C10—H10C	109.5
C2—C7—H7B	109.5	C9—N1—C1	120.87 (19)
			
C6—C1—C2—C3	1.9 (3)	C8—C4—C5—C6	−177.9 (2)
N1—C1—C2—C3	178.55 (19)	C2—C1—C6—C5	−0.9 (4)
C6—C1—C2—C7	−178.0 (2)	N1—C1—C6—C5	−177.5 (2)
N1—C1—C2—C7	−1.4 (3)	C4—C5—C6—C1	−0.7 (4)
C1—C2—C3—C4	−1.5 (3)	C9^i^—C9—N1—C1	178.3 (2)
C7—C2—C3—C4	178.4 (2)	C10—C9—N1—C1	−2.4 (3)
C2—C3—C4—C5	0.0 (3)	C6—C1—N1—C9	−78.8 (3)
C2—C3—C4—C8	179.0 (2)	C2—C1—N1—C9	104.6 (2)
C3—C4—C5—C6	1.1 (4)		

Symmetry codes: (i) −*x*+1, −*y*+2, −*z*+1.
